# Blood Biomarkers in Frontotemporal Dementia: Review and Meta-Analysis

**DOI:** 10.3390/brainsci11020244

**Published:** 2021-02-15

**Authors:** Sofia Ntymenou, Ioanna Tsantzali, Theodosis Kalamatianos, Konstantinos I. Voumvourakis, Elisabeth Kapaki, Georgios Tsivgoulis, George Stranjalis, George P. Paraskevas

**Affiliations:** 1 Department of Neurology, Evangelismos Hospital, 10676 Athens, Greece; 2 2^nd^ Department of Neurology, School of Medicine, “Attikon” University General Hospital, National and Kapodistrian University of Athens, 12462 Athens, Greece; docjo1989@gmail.com (I.T.); cvoumvou@otenet.gr (K.I.V.); tsivgoulisgiorg@yahoo.gr (G.T.); geoprskvs44@gmail.com (G.P.P.); 3 Department of Neurosurgery, School of Medicine, Evangelismos Hospital, National and Kapodistrian University of Athens, 10676 Athens, Greece; tkalamatian@med.uoa.gr (T.K.); stranjal@otenet.gr (G.S.); 4 Ward of Cognitive and Movement Disorders, 1^st^ Department of Neurology, School of Medicine, Eginition Hospital, National and Kapodistrian University of Athens, 11528 Athens, Greece; ekapaki@med.uoa.gr; 5 Unit of Neurochemistry and Biological Markers, Department of Neurology, School of Medicine, Eginition Hospital, National and Kapodistrian University of Athens, 11528 Athens, Greece

**Keywords:** blood biomarkers, frontotemporal dementia, progranulin

## Abstract

Biomarkers in cerebrospinal fluid (CSF) are useful in the differential diagnosis between frontotemporal dementia (FTD) and Alzheimer’s dementia (AD), but require lumbar puncture, which is a moderately invasive procedure that can cause anxiety to patients. Gradually, the measurement of blood biomarkers has been attracting great interest. Testing blood instead of CSF, in order to measure biomarkers, offers numerous advantages because it negates the need for lumbar puncture, it is widely available, and can be repeated, allowing the prediction of disease course. In this study, a systematic review of the existing literature was conducted, as well as meta-analysis with greater emphasis on the most studied biomarkers, p-tau and progranulin. The goal was to give prominence to evidence regarding the use of plasma biomarkers in clinical practice.

## 1. Introduction

Frontotemporal dementia (FTD) is a heterogeneous group of related conditions resulting from neurodegenerative disorders in the frontal and/or anterior temporal lobe of the brain [1]. Clinically, all these disorders may be presented as behavioral variant frontotemporal dementia (bvFTD) or primary progressive aphasia (PPA), whilst variants with parkinsonism and motor neuron disease are also recognized [2].

FTD is the third most common cause of neurodegenerative dementia, following Alzheimer’s disease (AD) and dementia with Lewy Bodies, and represents up to 20% of dementia cases of adults under 65 years old [1]. About 30–50% of cases with FTD are familial, 15% of which are inherited by an autosomal dominant pattern. Mutations in three genes have been associated with the majority of familial FTD: microtubule-associated protein τ *(MAPT*)-gene on chromosome 17, granulin (*GRN*)-gene on chromosome 17, and chromosome 9 open reading frame 72 *(C9orf72*) (the most common mutation, 12–15%) [3]. Other rare mutations include valosin-containing protein (VCP), charged multivesicular body protein 2B (CHMP2B), fused in sarcoma gene (*FUS*), TAR DNA-binding protein (*TARDBP*), integral membrane protein 2B (ITM2B) and tank-binding kinase 1 (*TBK*1) and TATA box binding protein (*TBP*), sequestosome 1 (*SQSTM1*), ubiquilin 2 (*UBQLN2*), coiled-coil-helix-coiled-coil-helix domain-containing protein 10 (*CHCHD10*), optineurin (*OPTN*) and cyclin F (*CCNF*). These rare mutations account for less than 5% of all FTD mutations and can be found in a limited number of families worldwide [4].

The differential diagnosis of frontotemporal dementia can be a real challenge. Neuroimaging, including magnetic resonance imaging (*MRI*), resting state functional MRI, 18F-fluorodeoxyglucose-positron emission tomography (*18F-FDG PET*) and 99mTechnetium-hexamethylpropanolamine oxime single photon emission computed tomography (*HMPAO-SPECT*), may be helpful in the diagnostic process [5]. However, findings may not be specific to FTD. Cerebrospinal fluid (CSF) biomarkers such as phosphorylated tau (p-Tau), total tau (T-tau), amyloid-β peptide 1–42 (Aβ42) can be very useful in the differential diagnosis, by excluding AD, but lumbar puncture is a moderately invasive procedure that can cause anxiety to patients [6]. This leads to an increasing need for the identification of plasma biomarkers, which through a less invasive procedure may prove useful in the routine clinical practice.

## 2. Materials and Methods

This systematic review was conducted following the PRISMA statement. We screened PubMed and Medline search engines for articles published during the last 15 years, together with references from the included studies. The research consisted of the following term combinations: “dementia” OR “frontotemporal” OR “FTD” OR “Alzheimer” OR “neurodegeneration” AND “p-tau” OR “phosphotau” OR “progranulin” OR “biomarker” OR “plasma biomarker” OR “blood biomarker” OR “serum biomarker”, and yielded 12,805 results. Only clinical studies, clinical trials and randomized controlled trials were included in the research, narrowing the results down to 604. Animal trials were excluded.

Separately, we searched for the terms “frontotemporal dementia”, “progranulin” and “phospho-Tau”. All types of articles of the last 10 years were deemed eligible for inclusion.

### 2.1. Inclusion Criteria

We included studies that measured blood biomarkers in patients with FTD compared to patients with AD or mild cognitive impairment or any other type of dementia as well as control groups. Studies measuring biomarkers in family members of FTD patients also fulfilled the inclusion criteria. Furthermore, studies which compared blood biomarkers to CSF biomarkers in patients with FTD were also eligible for inclusion. The purpose of this study was to investigate biomarkers that may be useful in everyday clinical practice. Therefore, emphasis has been given to proteinaceous molecules. Other potential biomarkers are mentioned, but not thoroughly investigated.

### 2.2. Exclusion Criteria

We excluded reviews, case reports and animal trials.

### 2.3. Analysis

**Figure 1:**
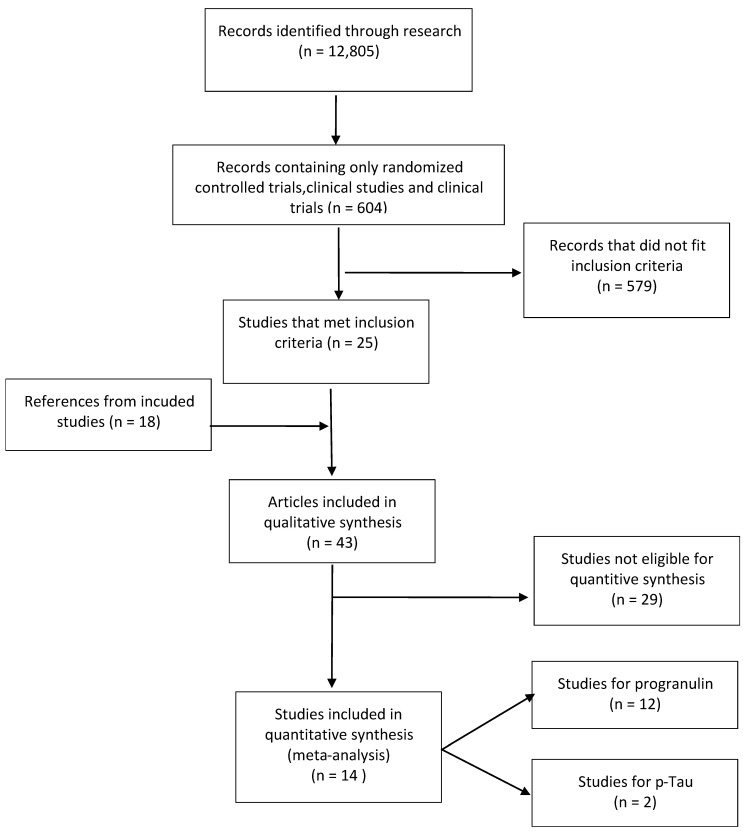
Flow diagram of collecting and evaluating research used in this review.

From the initial research of 604 results, 25 studies met the inclusion criteria. Additionally, 18 more articles, found as references from the included studies, were used in the qualitative synthesis of the review (Figure 1). From the articles included in the qualitative synthesis, 12 studies referred to progranulin, 2 studies referred to phosphorylated Tau(p-Tau), 4 studies referred to plasma Tau, 5 studies referred to neurofilament light chain protein (*NfL)*, 2 studies referred to transactive response DNA binding protein 43 (*TDP-43*), and 3 studies referred to glial fibrillary acidic protein (*GFAP*).

## 3. Statistical Analysis

Meta-analysis was performed following the random effects model with DerSimonian–Laird method, as performed by the RevMan software [7]. Although T^2^ and X^2^ were also reported, I^2^ was the most important measure of heterogeneity (low < 25%, moderate 25–75%, high > 75%). For publication bias, a funnel plot was created (Trim and Fill method), and Egger’s regression was performed by Meta-Essentials 1.5 (2020) [8]. Sensitivity analyses and subgroup analyses were also performed. When median and range were given, the mean and SD were calculated according to Hozo et al. 2005 [9]. However, when the median and interquartile range were given, mean and SD were calculated according to Wan X et al. 2014 [10].

## 4. Results

### 4.1. Biomarkers in FTD

Fluid biomarkers in frontotemporal dementia are measured in CSF and blood (plasma or serum). They can be used in differentiating FTD from Alzheimer’s disease (AD) or even identifying presymptomatic patients of familial cases. In CSF, neuronally derived molecules can be found at higher concentrations compared to blood. Blood, on the other hand, has increasingly attracted interest as a more accessible fluid alternative. Apart from diagnosis, blood biomarkers may be used as a screening tool for families of patients with familial FTD or to assess the effectiveness of a potential treatment [11].

### 4.2. Blood-Based Biomarkers in FTD

#### 4.2.1. Neurofilament Light Chain Protein (NfL)

Neurofilament light chain protein (NfL) has been measured in many studies as a CSF biomarker of FTD cases [12], and has been correlated to disease severity [13] (Table 1). Apart from CSF, NfL can be measured in serum with standard immunoassay format based on the single-molecule array (Simoa) technique that allows quantification to concentrations <1 pg/mL of the analyte [13].

Regarding studies measuring NfL in the different genotypes of FTD (GRN mutation, MAPT mutation and C9orf72mutation), NfL levels in patient-carriers are elevated compared to both the presymptomatic carriers and control groups [14]. Among the different clinical subtypes of FTD, there is evidence that NfL levels are increased in patients with the variant of motor neuron disease, and between PPA and bvFTD, patients with PPA have elevated NfL levels compared to bvFTD patients [15].

Studies display higher blood NfL levels in FTD patients and a correlation between serum levels of NfL and brain atrophy, suggesting longitudinal NfL measurements for mutation carrier identification and for disease progression [16], and suggesting longitudinal NfL measurements for mutation-carrier identification [17]. Another study relating plasma NfL to diffusion tensor imaging (DTI) metrics in patients with FTD, and specifically the bvFTD patients, displayed white matter involvement in FTD and provided further evidence that serum NfL may reflect the brain neurodegeneration process [18].

#### 4.2.2. Transactive Response DNA Binding Protein 43 (TDP-43)

Another biomarker that has been studied both in CSF and in blood is TDP-43. Cytoplasmic neuronal and glial inclusions of TDP-43 are commonly found in certain histological forms of frontotemporal lobar degeneration (FTLD), known as FTLD-ubiquitinated (FTLD-U), which are clinically expressed as FTD variants [22]. The existing studies measuring blood levels of TDP-43 in FTD and AD reveal increased blood levels in FTD patients compared to controls [23]. There is no specific evidence showing prevalence of TDP-43 levels in FTD compared to AD patients, probably due to the observation that a number of patients with AD have TDP-43 pathological changes within their brain [23]. A phosphorylated form of TDP-43 in plasma has been detected in higher levels in FTD patients compared to control groups, but data are still insufficient [24]. 

#### 4.2.3. Glial Fibrillary Acidic Protein (GFAP)

Glial fibrillary acidic protein (GFAP) is a measure of astrogliosis and has been used in many studies as a potential biomarker. Although the initial studies did not reveal encouraging results, one study of plasma GFAP in FTD-gene variants revealed an increased level of GFAP in GRN-mutation carriers than C9orf72 or MAPT mutations, and a significantly higher concentration in the symptomatic GRN mutation carriers versus the presymptomatic [25]. Among the clinical subtypes of FTD, a retrospective study measuring GFAP concentration revealed elevated plasma GFAP levels in all different subgroups compared to healthy controls, but no significant differences among the different subgroups [26]. Another multicentric study measuring both plasma and CSF biomarkers in late-onset and presenile bvFTD patients also demonstrates increased plasma GFAP levels in all bvFTD patients compared to controls, but without statistically important differences between the two groups [27].

#### 4.2.4. Plasma Tau

Plasma tau has been measured in many studies. Increased plasma total tau levels may be linked with cognitive impairment, but it is not related to AD or FTD [28]. A study including 176 participants, with different FTD variants, demonstrated increased plasma tau levels in all clinical FTD subgroups, but in regard to the genetic subtypes only in MAPT mutations [29].

#### 4.2.5. Progranulin

Progranulin is a glycoprotein coded by the GRN gene, on the chromosome 17q21. Among the most frequent genetic causes of FTD are the GRN mutations [30]. They are responsible for 20% of familial cases. The most pathogenic variants in GRN are mutations such as nonsense, frameshift, or splicing mutations, that cause haploinsufficiency leading to reduced levels of progranulin [31]. Progranulin can be measured either in CSF or in blood [32]. Many studies have measured plasma or serum progranulin, using ELISA methods, in patients with FTD both in carriers and in non-carriers of GRN mutations, as well as in presymptomatic carriers of GRN mutation and in control groups. Based on the existing results, progranulin levels seem to be a useful in identifying GRN mutation carriers from other mutation carriers such as C9orf72, according to a study performed in 2012 [33], or non-GRN mutation carriers (Table 2).

According to the literature, low levels of plasma progranulin can be used as an accurate method to discriminate GRN carriers from control groups with a very high sensitivity and specificity. Studies suggest different levels of blood progranulin as a cut-off in discriminating GRN mutations, with a more recent study rising the threshold level to 71.0 ng/mL.

Plasma progranulin levels have a wide range both in control groups and patient groups, leading to the question of whether other factors may contribute to regulating progranulin levels. Some studies suggest gender being a modifying factor, which has not been statistically proven. Age and body mass index do not influence plasma levels. On the other hand, autoimmune diseases, diabetes [34], cancer [35], autism [36], and several genetic variants such as rs5848 polymorphism located on GRN gene, transmembrane protein 106B (TMEM106B) and rs646776 polymorphism on sortilin (SORT1), have been suggested to affect progranulin levels [37]. Different genotypes of the same polymorphisms may also influence the progranulin levels. In particular, among the genotypes of rs5848 polymorphism, the TT genotype exhibits the lowest progranulin level followed by CT genotype and the CC genotype, which exhibits the highest level [37], whereas in the rs646776 polymorhism on SORT1, the CC genotype exhibits the lowest progranulin level followed by the CT genotype and the TT genotype, which exhibits the highest level [32].

#### 4.2.6. p-Tau

The differential diagnosis of FTD compared to AD has been based on the CSF biomarkers of Aβ42, total tau and p-Tau. In an effort to make biomarkers more accessible for clinical practice, apart from plasma total tau, blood p-Tau has also been studied in cognitive impaired patients (Table 3). Phosphorylated tau at threonine 181 (p-Tau181) has been measured in patients with cognitive impairment. Evidence shows that p-Tau181 is elevated in AD dementia compared to cognitive impaired patients (including different types of dementia) [48], making it a possible biomarker for differentiation between AD and FTD. A retrospective study published in 2020, including 404 participants, revealed that median plasma pTau181 concentrations were elevated in patients with AD compared to patients with FTLD [49]. In addition, pTau181 levels were associated with Aβ-PET and CSF pTau181, regardless of the diagnosis [49]. The difference in the levels of p-Tau181 between AD and FTD patients was also supported by another study that evaluated patients from two independent cohorts [50]. p-Tau181 levels do not have any correlation with age or gender. There are limited number of studies comparing p-Tau181 between AD and FTD. Apart from p-Tau181, p-Tau217 has also been investigated both in CSF [51] and in plasma [52]. From these findings, plasma p-Tau217 differentiated AD from FTD and other neurodegenerative diseases, and may show higher accuracy than p-Tau181.

#### 4.2.7. Other Possible Biomarkers

Other molecules that have been investigated as possible biomarkers include cytokines and chemokines, synaptic markers, and micro-RNA. Proinflammatory interleukins (ILs), such as IL-6 in plasma [41], IL-8 [53] or IL-15 in CSF, interferon gamma-induced protein 10 (IP-10) and tumor necrosis factor family cytokines (TNF-α) are some of the cytokines and chemokines that have already been measured in FTD patients [53]. A great number of studies have focused on micro-RNAs (mRNAs) in FTD, with their results showing a potential use of mRNAs both as a diagnostic tool and as a staging tool of FTD [54]. Additionally, synaptic markers such as synaptosomal-associated protein, 25 kDa (SNAP-25) and synaptotagmin [55], and neurotransmitters such as dopamine (DA) and its metabolite 3,4-dihydroxyphenylacetic acid (DOPAC), have also been investigated [56].

#### 4.2.8. Meta-Analysis

As already mentioned, the purpose of this study was to investigate biomarkers that may be useful in everyday clinical practice. For this reason, our meta-analysis focused on biomarkers that could be used as a differential tool of FTD vs. AD, or among genetic FTD subtypes. Therefore, only the studies of progranulin and p-Tau were processed with statistical analysis. Data from the existing studies of the other biomarkers did not fulfill our purpose.

**Figure 2:**
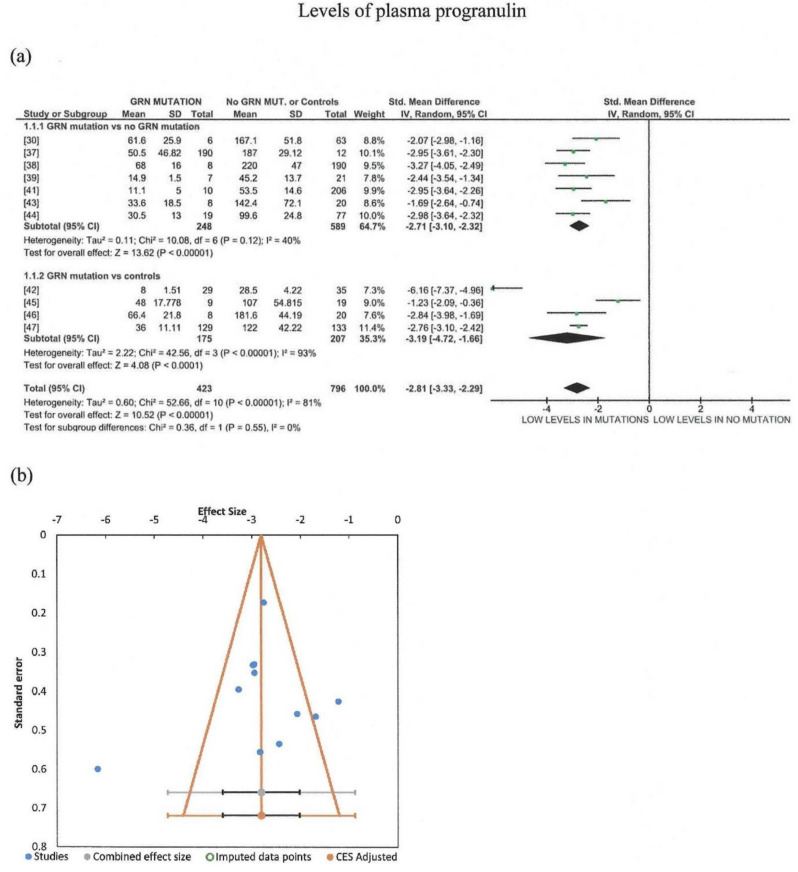
Meta-analysis (using the random effects model with DerSimonian–Laird method) of studies measuring plasma progranulin levels in carriers of GRN mutations vs. FTD with no GRN mutation or controls, with subgroup analysis (**a**). In the forest plot, the left side favors lower levels in the presence of GRN mutations, while the right side favors lower levels in the absence of GRN mutations or in controls. Effect sizes are represented as green squares with 95% confidence intervals. In the summary rows, the weighted average effect (or “combined” effect size) is represented as a diamond (**b**). A funnel plot of all 11 studies is included [30,37,38,39,41,42,43,44,45,46,47].

Furthermore, meta-analysis was conducted for studies regarding plasma progranulin levels in carriers of GRN mutations vs. FTD with no GRN mutation or controls. Only one study containing familial cases was included [40]. Through our meta-analysis, 1568 subjects were included, 423 of which were GRN mutation carriers, whereas 608 were non-carriers of GRN mutations, and 537 were controls. The random effects model was used, indicating that patients with GRN mutation had significantly lower levels of plasma progranulin as compared to FTD (Figure 2a). Significant heterogeneity of the studies was detected (I^2^ = 0.81), although all studies agreed that GRN mutations resulted in lower levels of plasma progranulin. Most of the studies measured progranulin by ELISA kits provided by Adipogen Inc (Seoul, South Korea) [30,37,38,40,43,45,46,47]. Sensitivity analysis revealed that the removal of studies using ELISA provided by R&D systems (Mineapolis USA) [39,44] and BioVendor (BioVendor (Brno, Czech Republic) [42], did not reduce heterogeneity. However, subgroup analysis revealed that when GRN mutation carriers were compared with no GRN mutations, heterogeneity was reduced to moderate, non-significant levels, while comparison with controls resulted in a very high heterogeneity. Testing for publication bias showed that three studies fell out of the funnel (Figure 2b); however, Egger’s test resulted in a nonsignificant result (*p* = 0.373).

**Figure 3:**
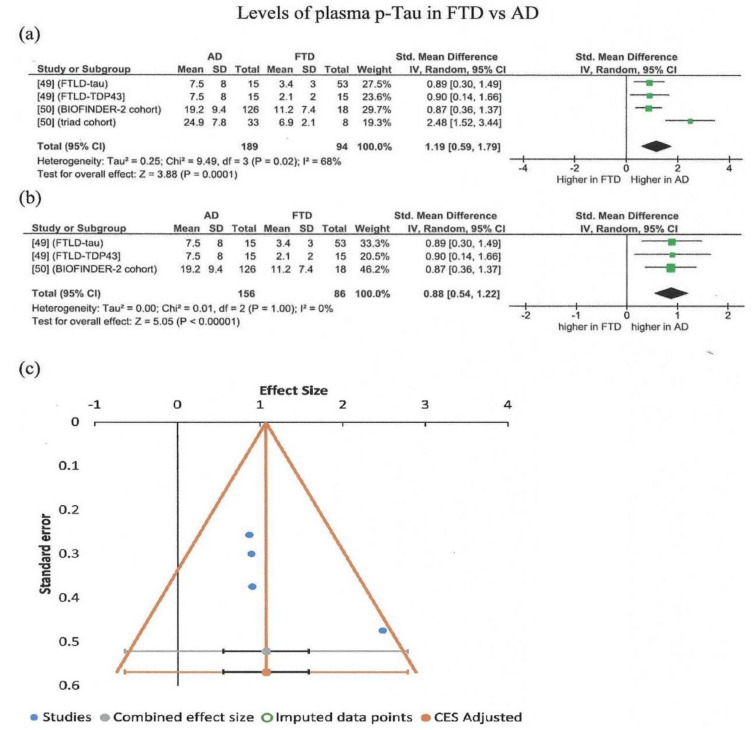
Meta-analysis (using the random effects model with DerSimonian–Laird method) of studies measuring p-Tau for all studies included (**a**) and after removal of the fourth study (triad cohort [50]) (**b**). In the forest plot, the right side favors higher levels of p-Tau in AD, while the left side favors higher values in FTD. Effect sizes are represented as green squares with 95% confidence intervals. In the summary row, the weighted average effect (or “combined” effect size) is represented as a diamond. (**c**) Funnel plot of all four studies (all studies are located within the “funnel”) [49,50].

Μeta-analysis was also conducted for studies regarding plasma p-Tau levels in AD vs. FTD. The total number of subjects included was 283 (four studies, FTD = 94, AD = 189). The random effects model was used, indicating that patients with AD have significantly higher levels of p-Tau181 as compared to FTD (Figure 3a). Moderate heterogeneity of the studies was detected (I^2^ = 0.68), although all studies agreed that AD showed higher p-Tau181 levels as compared to FTD. Sensitivity analysis showed that removal of the fourth study (triad cohort, different population [50]), also resulted in a significant overall effect, but with no heterogeneity (Figure 3b). No significant publication bias was observed, by visual inspection of the funnel plot of all four studies (Figure 3c). Egger’s test was not meaningful because few studies were included (fewer than 10).

## 5. Discussion

A number of studies show promising results in using blood biomarkers for the (differential) diagnostic workup of patients with FTD in everyday practice. As a result, it is worth exploring whether these biomarkers could be utilized in the treatment decision process.

As already mentioned, NfL levels have been correlated to disease severity and brain atrophy. Monitoring NfL levels in patients with FTD as the disease progresses as well as comparing NfL levels among FTD patients of different stages may lead to the future recognition of NfL as a useful biomarker not only for the differential process of FTD but also for the disease intensity.

TDP-43 has also been suggested as a potential biomarker for the discrimination of patients with FTD or AD with TDP-43 pathology from those without TDP-43 pathology, but the existing evidence is insufficient. Measuring blood TDP-43 may not be useful in differentiating different types of dementia but may become valuable if TDP-43-based treatments become available.

In addition, GFAP seems to be a potential biomarker of FTD patients, especially for GRN mutation carriers. Given the fact that the symptomatic GRN mutation carriers have higher concentration levels versus presymptomatic patients, GFAP could provide a prognostic tool to symptom onset.

Progranulin levels in blood have been assessed in patients with FTD carrying the GRN mutations, patients with FTD without GRN mutations, and in control groups. Furthermore, progranulin levels in GRN mutation carriers are significantly lower compared to both non-carriers and the control group, making progranulin a useful biomarker in discriminating GRN mutation carriers. There is also much evidence that this protein could be used instead of molecular genetic analysis to discriminate GRN carriers from non-carriers in families with FTD patients.

Different cut-off levels are proposed from the studies, with the most recent proposing a threshold of 71ng/mL. Although there are studies displaying indications about gender influence on the progranulin level, this has not been statistically supported.

From the current studies, patients with AD have increased p-Tau levels compared to both the FTD patients and the control group. Comparing subjects with cognitive impairment—both FTD and AD groups—to control groups, a weak correlation of cognitive impairment to p-Tau levels was observed. Moreover, mean values of p-Tau were higher in the cognitive-impaired groups compared to control groups, but among the cognitive impaired subjects only AD patients had significantly higher p-Tau levels. This evidence suggests that p-Tau could be a useful tool in differentiating FTD from AD. p-Tau217 may have higher accuracy than p-Tau181, but more research needs to be conducted.

## 6. Conclusions

There is a great variety of blood biomarkers studied in FTD, some more promising than others. The future in biomarkers is finding sensitive molecules that will differentiate the FTD variants, predict disease intensity, or even assist the treatment choice. More trials with larger sample collection are yet to be performed in order to fulfill this purpose.

## Figures and Tables

**Table 1:** Studies measuring Neurofilament light chain protein (NfL) levels.

	Frontotemporal Dementia (FTD) Patients	Alzheimer’s Disease (AD) Patients	Control Group
[16] median (IQR)	52 (24–69) pg/mL (*n* = 59)	-	8 pg/mL (6–11) (*n* = 127)
[17] (mean ± SD)	77.9 ± 51.3 pg/mL (*n* = 67)	-	19.6 ± 8.2 pg/mL (*n* = 28)
[19] median (IQR)	46.5 (19.4–103) pg/mL (*n* = 12)	38.6 (21.6–240) pg/mL (*n* = 20)	27.3 (0.7–210) pg/mL (*n* = 44)
[20] median (IQR)	47.2 (26.9–76.6) pg/mL (*n* = 41)	-	17.8 (7.7–30.7) pg/mL (*n* = 46)
[21] Median (IQR)	49.0 (35.2) pg/mL (*n* = 74)	32.3 (15.8) pg/mL (*n* = 26)	21.7 (20.6) pg/mL (*n* = 15)

**Table 2:** Studies measuring progranulin levels.

	GRN Mutations	No GRN Mutations	Control Group	Threshold
[38] mean ± SD	68 ± 16 ng/mL (*n* = 8)	220 ± 47 ng/mL (*n* = 190)	228 ± 50 ng/mL (*n* = 70)	112 ng/mL
[37] mean 95%CI	50.5 (43.8–57.2) ng/mL (*n* = 190)	187 (167–204) ng/mL (*n* = 12)	195 (180–211) ng/mL (*n* = 36)	-
[30] mean ± SD	61.6 ± 25.9 ng/mL (n = 6)	167.1 ± 51.8 ng/mL (*n* = 63)	-	110.9 ng/mL
[39] mean ± SD	14.9 ± 1.5 ng/mL (*n* = 7)	45.2 ± 13.7 ng/mL (*n* = 21)	44.1 ± 9.8 ng/mL (*n* = 62)	23.6 ng/mL
[40] mean ± SD	47.7 ng/mL (*n* = 1)	162.4 ± 69.4 ng/mL (*n* = 21)	137.6 ± 34.2 ng/mL (*n* = 17)	-
[41] mean ± SD	11.1 ± 5 ng/mL (*n* = 10)	53.5 ± 14.6 ng/mL (*n* = 206)	53.7 ± 13.7 ng/mL (*n* = 161)	22 ng/mL
[42] median (range)	8 (5.2–11.3) ng/mL (*n* = 29)	-	28.5 (21.5–39.2) ng/mL (*n* = 35)	-
[43] mean ± SD	33.6 ± 18.5 ng/mL (*n* = 8)	142.4 ± 72.1 ng/mL (*n* = 20)	-	-
[44] mean ± SD	30.5 ± 13 ng/mL (*n* = 19)	99.6 ± 24.8 ng/mL (*n* = 77)	-	61.55 ng/mL
[45] median (IQR)	48 (39–63) ng/mL (*n* = 9)	107 (91–125) ng/mL (*n* = 19)	130 (101–175) ng/mL (*n* = 20)	75.3 ng/mL
[46] mean (range)	66.4 (23–85.1) ng/mL (*n* = 8)	-	181.6 (101.1–266) ng/mL (*n* = 20)	93.1 ng/mL
[47] 25th–75th percentiles	36 (29–44) ng/mL (*n* = 129)	-	122 (101–158) ng/mL (*n* = 133)	71 ng/ml

**Table 3:** Studies measuring p-Tau.

	FTD Patients	AD Patients	Control Group
[50] Triad cohort (mean ± SD)	6.9 ± 2.1 pg/mL (*n* = 8)	24.9 ± 7.8 pg/mL (*n* = 33)	10 ± 3.3 pg/mL (*n* = 113)
[50] BIOFINDER-2Cohort (mean ± SD)	11.2 ± 7.4 pg/mL (*n* = 18)	19.2 ± 9.4 pg/mL (*n* = 126)	9.4 ± 6 pg/mL (*n* = 337)
[49] (mean ± SD)	FTLD-tau: 3.4 ± 3 pg/Ml (*n* = 53)	7.5 ± 8 pg/mL (*n* = 15)	2.0 ± 2 pg/mL (*n* = 44)
[49] (mean ± SD)	FTLD-TDP:2.1 ± 2 pg/mL (*n* = 15)	7.5 ± 8 pg/mL (*n* = 15)	2.0 ± 2 pg/mL (*n* = 44)

## Data Availability

Not applicable.

## References

[1] Bang J., Spina S., Miller B.L. (2015). Frontotemporal dementia. Lancet.

[2] Lanata S.C., Miller B.L. (2016). The behavioural variant frontotemporal dementia (bvFTD) syndrome in psychiatry. J. Neurol. Neurosurg. Psychiatry.

[3] Sieben A., Van Langenhove T., Engelborghs S., Martin J.-J., Boon P., Cras P., De Deyn P.-P., Santens P., Van Broeckhoven C., Cruts M. (2012). The genetics and neuropathology of frontotemporal lobar degeneration. Acta Neuropathol..

[4] Greaves C.V., Rohrer J.D. (2019). An update on genetic frontotemporal dementia. J. Neurol..

[5] Bruun M., Koikkalainen J., Rhodius-Meester H.F., Baroni M., Gjerum L., Van Gils M., Soininen H., Remes A.M., Hartikainen P., Waldemar G. (2019). Detecting frontotemporal dementia syndromes using MRI biomarkers. NeuroImage: Clin..

[6] Sancesario G.M., Bernardini S. (2015). How many biomarkers to discriminate neurodegenerative dementia?. Crit. Rev. Clin. Lab. Sci..

[7] (2020). Review Manager (RevMan) [Computer Program].

[8] Suurmond R., Van Rhee H., Hak T. (2017). Introduction, comparison, and validation ofMeta-Essentials: A free and simple tool for meta-analysis. Res. Synth. Methods.

[9] Hozo S.P., Djulbegovic B., Hozo I. (2005). Estimating the mean and variance from the median, range, and the size of a sample. BMC Med. Res. Methodol..

[10] Wan X., Wang W., Liu J., Tong T. (2014). Estimating the sample mean and standard deviation from the sample size, median, range and/or interquartile range. BMC Med. Res. Methodol..

[11] Zetterberg H., Van Swieten J.C., Boxer A.L., Rohrer J.D. (2018). Review: Fluid biomarkers for frontotemporal dementias. Neuropathol. Appl. Neurobiol..

[12] Abu-Rumeileh S., Steinacker P., Polischi B., Mammana A., Bartoletti-Stella A., Oeckl P., Baiardi S., Zenesini C., Huss A., Cortelli P. (2020). CSF biomarkers of neuroinflammation in distinct forms and subtypes of neurodegenerative dementia. Alzheimer’s Res. Ther..

[13] Scherling C.S., Hall T., Berisha F., Klepac K., Karydas A., Coppola G., Kramer J.H., Rabinovici G., Ahlijanian M., Miller B.L. (2014). Cerebrospinal fluid neurofilament concentration reflects disease severity in frontotemporal degeneration. Ann. Neurol..

[14] Rohrer J.D., Woollacott I.O., Dick K.M., Brotherhood E., Gordon E., Fellows A., Toombs J., Druyeh R., Cardoso M.J., Ourselin S. (2016). Serum neurofilament light chain protein is a measure of disease intensity in frontotemporal dementia. Neurology.

[15] an Der Ende E.L., Meeter L.H., Poos J.M., Panman J.L., Jiskoot L.C., Dopper E.G.P., Papma J.M., De Jong F.J., Verberk I.M.W., Teunissen C. (2019). Serum neurofilament light chain in genetic frontotemporal dementia: A longitudinal, multicentre cohort study. Lancet Neurol..

[16] Neumann M., Sampathu D.M., Kwong L.K., Truax A.C., Micsenyi M.C., Chou T.T., Bruce J., Schuck T., Grossman M., Clark C.M. (2006). Ubiquitinated TDP-43 in Frontotemporal Lobar Degeneration and Amyotrophic Lateral Sclerosis. Science.

[17] Foulds P., McAuley E., Gibbons L., Davidson Y., Pickering-Brown S.M., Neary D., Snowden J.S., Allsop D., Mann D.M.A. (2008). TDP-43 protein in plasma may index TDP-43 brain pathology in Alzheimer’s disease and frontotemporal lobar degeneration. Acta Neuropathol..

[18] Foulds P.G., Davidson Y., Mishra M., Hobson D.J., Humphreys K.M., Taylor M., Johnson N., Weintraub S., Akiyama H., Arai T. (2009). Plasma phosphorylated-TDP-43 protein levels correlate with brain pathology in frontotemporal lobar degeneration. Acta Neuropathol..

[19] Heller C., Foiani M.S., Moore K., Convery R., Bocchetta M., Neason M., Cash D.M., Thomas D., Greaves C.V., Woollacott I.O. (2020). Plasma glial fibrillary acidic protein is raised in progranulin-associated frontotemporal dementia. J. Neurol. Neurosurg. Psychiatry.

[20] Yang S.-Y., Chiu M.-J., Chen T.-F., Lin C.-H., Jeng J.-S., Tang S.-C., Lee Y.-F., Yang C.-C., Liu B.-H., Chen H.-H. (2017). Analytical performance of reagent for assaying tau protein in human plasma and feasibility study screening neurodegenerative diseases. Sci. Rep..

[21] Foiani M.S., Woollacott I.O., Heller C., Bocchetta M., Heslegrave A., Dick K.M., Russell L.L., Marshall C.R., Mead S., Schott J.M. (2018). Plasma tau is increased in frontotemporal dementia. J. Neurol. Neurosurg. Psychiatry.

[22] Ghidoni R., Paterlini A., Benussi L. (2012). Circulating progranulin as a biomarker for neurodegenerative diseases. Am. J. Neurodegener. Dis..

[23] Olszewska D.A., Lonergan R., Fallon E.M., Lynch T. (2016). Genetics of Frontotemporal Dementia. Curr. Neurol. Neurosci. Rep..

[24] Nicholson A.M., Finch N.A., Thomas C.S., Wojtas A., Rutherford N.J., Mielke M.M., Roberts R.O., Boeve B.F., Knopman D.S., Petersen R.C. (2014). Progranulin protein levels are differently regulated in plasma and CSF. Neurology.

[25] Jian J., Li G., Hettinghouse A., Liu C. (2018). Progranulin: A key player in autoimmune diseases. Cytokine.

[26] Arechavaleta-Velasco F., Perez-Juarez C.E., Gerton G.L., Diaz-Cueto L. (2017). Progranulin and its biological effects in cancer. Med. Oncol..

[27] Al-Ayadhi L.Y., A Mostafa G. (2011). Low plasma progranulin levels in children with autism. J. Neuroinflamm..

[28] Hsiung G.-Y.R., Fok A., Feldman H.H., Rademakers R., MacKenzie I.R. (2011). rs5848 polymorphism and serum progranulin level. J. Neurol. Sci..

[29] Janelidze S., Mattsson N., Palmqvist S., Smith R., Beach T.G., Serrano G.E., Chai X., Proctor N.K., Eichenlaub U., Zetterberg H. (2020). Plasma P-tau181 in Alzheimer’s disease: Relationship to other biomarkers, differential diagnosis, neuropathology and longitudinal progression to Alzheimer’s dementia. Nat. Med..

[30] Thijssen E.H., La Joie R., Wolf A., Strom A., Wang P., Iaccarino L., Bourakova V., Cobigo Y., Heuer H., Spina S. (2020). Diagnostic value of plasma phosphorylated tau181 in Alzheimer’s disease and frontotemporal lobar degeneration. Nat. Med..

[31] Karikari T.K., Pascoal T.A., Ashton N.J., Janelidze S., Benedet A.L., Rodriguez J.L., Chamoun M., Savard M., Kang M.S., Therriault J. (2020). Blood phosphorylated tau 181 as a biomarker for Alzheimer’s disease: A diagnostic performance and prediction modelling study using data from four prospective cohorts. Lancet Neurol..

[32] Janelidze S., Stomrud E., Smith R., Palmqvist S., Mattsson N., Airey D.C., Proctor N.K., Chai X., Shcherbinin S., Sims J.R. (2020). Cerebrospinal fluid p-tau217 performs better than p-tau181 as a biomarker of Alzheimer’s disease. Nat. Commun..

[33] Palmqvist S., Janelidze S., Quiroz Y.T., Zetterberg H., Lopera F., Stomrud E., Su Y., Chen Y., Serrano G.E., Leuzy A. (2020). Discriminative Accuracy of Plasma Phospho-tau217 for Alzheimer Disease vs Other Neurodegenerative Disorders. JAMA.

[34] Gibbons L., Rollinson S., Thompson J.C., Robinson A., Davidson Y.S., Richardson A., Neary D., Pickering-Brown S.M., Snowden J.S., Mann D.M. (2015). Plasma levels of progranulin and interleukin-6 in frontotemporal lobar degeneration. Neurobiol. Aging.

[35] Galimberti D., Schoonenboom N., Scheltens P., Fenoglio C., Venturelli E., Pijnenburg Y., Bresolin N., Scarpini E. (2006). Intrathecal chemokine levels in Alzheimer disease and frontotemporal lobar degeneration. Neurol..

[36] Grasso M., Piscopo P., Talarico G., Ricci L., Crestini A., Tosto G., Gasparini M., Bruno G., Denti M.A., Confaloni A. (2019). Plasma microRNA profiling distinguishes patients with frontotemporal dementia from healthy subjects. Neurobiol. Aging.

[37] Goetzl E.J., Kapogiannis D., Schwartz J.B., Lobach I.V., Goetzl L., Abner E.L., Jicha G.A., Karydas A.M., Boxer A., Miller B.L. (2016). Decreased synaptic proteins in neuronal exosomes of frontotemporal dementia and Alzheimer’s disease. FASEB J..

[38] Janssens J., Vermeiren Y., Van Faassen M., Van Der Ley C., Kema I.P., De Deyn P.P. (2020). Monoaminergic and Kynurenergic Characterization of Frontotemporal Dementia and Amyotrophic Lateral Sclerosis in Cerebrospinal Fluid and Serum. Neurochem. Res..

[39] Almeida M.R., Baldeiras I., Ribeiro M.H., Santiago B., Machado C., Massano J., Guimarães J., Oliveira C.R., Santana I. (2013). Progranulin Peripheral Levels as a Screening Tool for the Identification of Subjects with Progranulin Mutations in a Portuguese Cohort. Neurodegener. Dis..

[40] Finch N., Baker M., Crook R., Swanson K., Kuntz K., Surtees R., Bisceglio G., Rovelet-Lecrux A., Boeve B., Petersen R.C. (2009). Plasma progranulin levels predict progranulin mutation status in frontotemporal dementia patients and asymptomatic family members. Brain.

[41] Feneberg E., Steinacker P., Volk A.E., Weishaupt J.H., Wollmer M.A., Boxer A., Tumani H., Ludolph A.C., Otto M. (2015). Progranulin as a candidate biomarker for therapeutic trial in patients with ALS and FTLD. J. Neural Transm..

[42] Meeter L.H., Patzke H., Loewen G., Dopper E.G., Pijnenburg Y.A., Van Minkelen R., Van Swieten J.C. (2016). Progranulin Levels in Plasma and Cerebrospinal Fluid in Granulin Mutation Carriers. Dement. Geriatr. Cogn. Disord. Extra.

[43] Moretti D.V., Ebenussi L., Efostinelli S., Eciani M., Ebinetti G., Eghidoni R. (2016). Progranulin Mutations Affects Brain Oscillatory Activity in Fronto-Temporal Dementia. Front. Aging Neurosci..

[44] Galimberti D., Fumagalli G.G., Fenoglio C., Cioffi S.M., Arighi A., Serpente M., Borroni B., Padovani A., Tagliavini F., Masellis M. (2018). Progranulin plasma levels predict the presence of GRN mutations in asymptomatic subjects and do not correlate with brain atrophy: Results from the GENFI study. Neurobiol. Aging.

[45] Guven G., Bilgic B., Tufekcioglu Z., Unaltuna N.E., Hanagasi H., Gurvit H., Singleton A., Hardy J., Emre M., Gulec C. (2019). Peripheral GRN mRNA and Serum Progranulin Levels as a Potential Indicator for Both the Presence of Splice Site Mutations and Individuals at Risk for Frontotemporal Dementia. J. Alzheimer’s Dis..

[46] Sellami L., Rucheton B., Ben Younes I., Camuzat A., Saracino D., Rinaldi D., Epelbaum S., Azuar C., Levy R., Auriacombe S. (2020). Plasma progranulin levels for frontotemporal dementia in clinical practice: A 10-year French experience. Neurobiol. Aging.

[47] Goossens J., Bjerke M., Van Mossevelde S., Bossche T.V.D., Goeman J., De Vil B., Sieben A., Martin J.-J., Cras P., De Deyn P.P. (2018). Diagnostic value of cerebrospinal fluid tau, neurofilament, and progranulin in definite frontotemporal lobar degeneration. Alzheimer’s Res. Ther..

[48] Wilke C., Preische O., Deuschle C., Roeben B., Apel A., Barro C., Maia L., Maetzler W., Kuhle J., Synofzik M. (2016). Neurofilament light chain in FTD is elevated not only in cerebrospinal fluid, but also in serum. J. Neurol. Neurosurg. Psychiatry.

[49] Verde F., Steinacker P., Weishaupt J.H., Kassubek J., Oeckl P., Halbgebauer S., Tumani H., Arnim C.A.F.V., Dorst J., Feneberg E. (2019). Neurofilament light chain in serum for the diagnosis of amyotrophic lateral sclerosis. J. Neurol. Neurosurg. Psychiatry.

[50] Steinacker P., Anderl-Straub S., Diehl-Schmid J., Semler E., Uttner I., Von Arnim C.A., Barthel H., Danek A., Fassbender K., Fliessbach K. (2018). Serum neurofilament light chain in behavioral variant frontotemporal dementia. Neurology.

[51] Meeter L.H., Dopper E.G., Jiskoot L.C., Sanchez-Valle R., Graff C., Benussi L., Ghidoni R., Pijnenburg Y.A., Borroni B., Galimberti D. (2016). Neurofilament light chain: A biomarker for genetic frontotemporal dementia. Ann. Clin. Transl. Neurol..

[52] Cajanus A., Katisko K., Kontkanen A., Jääskeläinen O., Hartikainen P., Haapasalo A., Herukka S., Vanninen R., Solje E., Hall A. (2020). Serum neurofilament light chain in FTLD: Association with C9orf72, clinical phenotype, and prognosis. Ann. Clin. Transl. Neurol..

[53] Spotorno N., Lindberg O., Nilsson C., Waldö M.L., Van Westen D., Nilsson K., Vestberg S., Englund E., Zetterberg H., Blennow K. (2020). Plasma neurofilament light protein correlates with diffusion tensor imaging metrics in frontotemporal dementia. PLoS ONE.

[54] Benussi A., Ashton N.J., Karikari T.K., Gazzina S., Premi E., Benussi L., Ghidoni R., Rodriguez J.L., Emeršič A., Binetti G. (2020). Serum Glial Fibrillary Acidic Protein (GFAP) Is a Marker of Disease Severity in Frontotemporal Lobar Degeneration. J. Alzheimer’s Dis..

[55] Marelli C., Hourregue C., Gutierrez L.-A., Paquet C., De Champfleur N.M., De Verbizier D., Jacob M., Dubois J., Maleska A.M., Hirtz C. (2020). Cerebrospinal Fluid and Plasma Biomarkers do not Differ in the Presenile and Late-Onset Behavioral Variants of Frontotemporal Dementia. J. Alzheimer’s Dis..

[56] Dols-Icardo O., Suárez-Calvet M., Hernández I., Amer G., Antón-Aguirre S., Alcolea D., Fortea J., Boada M., Tárraga L., Blesa R. (2012). Expansion Mutation in C9ORF72 Does Not Influence Plasma Progranulin Levels in Frontotemporal Dementia. Neurobiol. Aging.

